# Testosterone Therapy on Active Surveillance and Following Definitive Treatment for Prostate Cancer

**DOI:** 10.1007/s11934-017-0695-6

**Published:** 2017-06-06

**Authors:** Vishnukamal Golla, Alan L. Kaplan

**Affiliations:** Department of Urology, David Geffen School of Medicine at UCLA, 10833 Le Conte Ave., Box 951738, Los Angeles, CA 90095-1738 USA

**Keywords:** Prostate cancer, Testosterone replacement therapy, Hypogonadism, Active surveillance, Testosterone, Testosterone deficiency

## Abstract

**Purpose of Review:**

Previously considered an absolute contraindication, the use of testosterone therapy in men with prostate cancer has undergone an important paradigm shift. Recent data has changed the way we approach the treatment of testosterone deficiency in men with prostate cancer. In the current review, we summarize and analyze the literature surrounding effects of testosterone therapy on patients being treated in an active surveillance protocol as well as following definitive treatment for prostate cancer.

**Recent Findings:**

The conventional notion that defined the relationship between increasing testosterone and prostate cancer growth was based on limited studies and anecdotal case reports. Contemporary evidence suggests testosterone therapy in men with testosterone deficiency does not increase prostate cancer risk or the chances of more aggressive disease at prostate cancer diagnosis. Although the studies are limited, men who received testosterone therapy for localized disease did not have higher rates of recurrences or worse clinical outcomes. Current review of the literature has not identified adverse progression events for patients receiving testosterone therapy while on active surveillance/watchful waiting or definitive therapies.

**Summary:**

The importance of negative effects of testosterone deficiency on health and health-related quality of life measures has pushed urologists to re-evaluate the role testosterone plays in prostate cancer. This led to a paradigm shift that testosterone therapy might in fact be a viable option for a select group of men with testosterone deficiency and a concurrent diagnosis of prostate cancer.

## Introduction

Dr. Charles Huggins described the relationship between serum testosterone and prostate cancer more than 70 years ago [1]. It is estimated that testosterone deficiency (TD) affects up to 25% of men over 40 with prevalence increasing significantly with age. Treatment for testosterone deficiency primarily involves exogenous testosterone supplementation via topical gels or liquids, implantable pellets, or injectable preparation. Testosterone therapy affords symptomatic benefits (e.g., increased energy and libido) as well reductions in body mass index (BMI), improved glycemic control and lipid profile, and improvement in bone mineralization and cardiovascular outcomes [2]*.* Despite growing awareness of the potential health benefits of testosterone therapy for men with TD, those with concurrent or historical prostate cancer are frequently denied exogenous testosterone therapy.

Until recently, conventional thinking dictated that testosterone therapy is an absolute contraindication in men with prostate cancer. However, a preponderance of new data has shed light on the significant benefits of testosterone on quality of life and the function of multiple organ systems. As a result, urologists are re-examining these historical assumptions regarding the effect of exogenous androgen on the prostate, especially in men diagnosed with prostate cancer.

New data published within the past decade has raised serious doubts about the risk of cancer progression among men with prostate cancer treated with testosterone therapy. However, physicians continue to have deep-seated fears that testosterone therapy can lead to prostate cancer recurrence or even rapid progression in men despite definitive treatment. There are fears that testosterone therapy can even unmask occult prostate cancer. Prostate cancer is the most commonly diagnosed malignancy in men after skin cancer with about 161, 000 new cases every year [3]. At the same time, the propensity for active surveillance has increased in the past decade with surveillance rates quoted as high as 64% at some urological practices [4]. Therefore, it is paramount that we critically evaluate the data on testosterone therapy and prostate cancer.

## Material and Method

We conducted a Medline and PubMed search from 1935 to 2017 to identify all publications related to the use of testosterone in men with prostate cancer, treated or otherwise. Original studies and review articles were included. Key words used for the search were “prostate cancer,” “testosterone replacement therapy,” “hypogonadism,” “active surveillance,” “testosterone,” “testosterone deficiency,” “testosterone therapy,” “radical prostatectomy,” and “radiation therapy.”

### Historical Perspective

These historical assumptions date back to the 1940s based on the clinical observations by Dr. Huggins and Dr. Hodges that prostate cancer was androgen dependent and that androgen deprivation resulted in the regression of prostate cancer with a concurrent drop in prostate-specific antigen (PSA). It was their seminal research in men with metastatic prostatic cancer that established the androgen hypothesis: that there is a direct correlation between androgen activity in the body and prostate cancer growth and development. This conclusion led to axiomatic teaching that administration of exogenous testosterone to men with prostate cancer would promote malignant prostate cell proliferation and progression.

In a 1935 research study, Kutscher and Wolbergs found that acid phosphatase was present in higher concentrations in human and monkey prostatic tissue than other tissues in the body [5]*.* In 1941, Huggins and Hodges investigated the effects of hormone manipulation in prostate cancer. In a three-patient case series, they showed prostate cancer regression following bilateral orchiectomy. Huggins and Hodges also showed that in men with metastatic prostate cancer treated with orchiectomy or with estrogen treatment, there was a demonstrative decrease in serum acid phosphatase activity [6]. Subsequent injection of exogenous testosterone propionate caused an increase in acid phosphatase above preinjection levels. And with cessation of injections, PAP levels would return to their baseline. This led to three important conclusions by Huggins and Hodges: (1) androgen activity in the body affected prostate cancer, (2) metastatic prostate cancer regressed with lower testosterone levels, and (3) prostate cancer is activated by androgen injections [7]. These studies formed the nuclear spark behind the standard of care in treating metastatic prostate cancer by establishing a direct correlation between testosterone levels and prostate cancer. Further in vitro and clinical work in that time period went on to suggest that exogenous administration of testosterone stimulated prostatic cancer cell growth. Therein lies the crux of the axiomatic teaching that exogenous testosterone should be avoided in men with prostate cancer.

## Testosterone Physiology and Its Role in Prostate Cancer

The majority of testosterone (90%) is synthesized by testicular Leydig cells (90%) under the control of the luteinizing hormone (LH) released from the anterior pituitary. The remaining 10% of testosterone is produced by the adrenal glands. Testosterone is the principal circulating androgen in men and is synthesized from a 27-carbon cholesterol that is altered in an enzymatic pathway to a 19-carbon steroid, a precursor to androgens. About 2% of testosterone is metabolically active and circulates within the serum in a free form or bound to the protein albumin (38%). Sixty percent of testosterone is bound to the sex hormone-binding globulin (SHBG), rendering it unavailable to most tissues. As men age, the amount of SHBG increases and concurrently the hypothalamus decreases the production of gonadotropins. This is one explanation for why TD is more prevalent in older men with prostate cancer.

Testosterone acts on prostatic epithelial cells by diffusing through the cellular membrane where it is then converted to 5alpha-dihydrotestosterone (DHT) by the enzyme 5-alpha reductase. Testosterone and DHT both act on the androgen receptor. The two main androgen receptor binding properties that regulate testosterone binding include receptor affinity and the intracellular concentration of androgens. In the prostate specifically, anything above a baseline serum testosterone concentration will play no role in further stimulating growth. This is because at this point all intraprostatic AR sites are completely bound [8•].

## Testosterone Deficiency

Testosterone deficiency is a clinical syndrome consisting of a several characteristic signs and symptoms combined with a low serum testosterone concentration (testosterone <300 ng/dL). Common symptoms include low libido, erectile dysfunction, reduced morning erections, depression, and fatigue. Clinical signs include anemia, decreased muscle mass, increased fat mass, and reduced bone mineral density. The literature reports varying prevalence estimates (7–18.4%), but in general, TD is common in adult men and increases with age [9]. Exogenous administration of testosterone to replete androgen levels has been shown to mitigate the negative consequences of TD predominantly body composition, metabolic state, as well as sexual and psychological factors [10].

Although the historical androgen hypothesis has always purported linear relationship between prostatic cancer cell growth and serum androgen, this assumption has not been adequately supported. Large prospective studies have not shown a link between endogenous serum testosterone level and the risk of developing prostate cancer. Additionally, there is no known correlation between serum testosterone levels and prostate size or PSA level [8•, 11]. This data led to the development of the saturation model to help explain these contradictory observations.

## Testosterone Saturation Model

The androgen receptor saturation model, first proposed by Morgentaler in 2006, explains the minor impact of testosterone therapy in non-castrate men. The model addresses two seemingly contradictory proposals: (1) Both benign and malignant prostatic neoplasms are unaffected by varying serum androgen concentration in the normal concentration range and (2) that there is exponential sensitivity to a variation in the androgen concentration at the lower concentration. The saturation model proposes that a decrease in serum testosterone below the maximal androgen receptor-binding threshold can cause substantial changes in biology. However, once the androgen receptor is saturated (approximately 4 nmol/L in vitro), any additional testosterone results in insignificant stimulation of the androgen receptor [7]. Marks et al. further supported the model by showing that when patients were treated with testosterone therapy, resulting in large increase in serum testosterone, androgen concentration in prostatic tissue remained unchanged. In addition, there was no effect on prostate tissue histology, tissue biomarkers, gene expression, and cancer incidence or severity [12].

The saturation point at which increasing serum T concentration does not cause any further appreciable growth is at approximately 240–250 ng/dL. This is in line with the numbers predicted for maximal androgen receptor binding to androgen (~240 ng/dL). Several studies showed that a baseline serum T concentration greater than 250 ng/dL did not demonstrate a rise in PSA [13, 14 15•].

### Testosterone Therapy in Men with Prostate Cancer on Active Surveillance/Watchful Waiting

To date, no randomized controlled trials have investigated the risks of testosterone therapy in men with diagnosed but untreated prostate cancer. In our literature review, we identified a relevant meta-analysis, systematic reviews, and several observational retrospective studies that addressed this topic.

A study by Calof et al. in 2005 looked at 19 randomized trials identified from 1966 to 2004 using the inclusion criteria of testosterone therapy for >90 days in men >45 years old with TD. The authors concluded that rates of prostate cancer, PSA > 4 ng/mL, and abnormal prostate biopsies were not significantly different between the testosterone therapy and the placebo cohort [16].

This data was corroborated by a systematic review done by Shabsigh et al. in 2009 examining 197 articles covering testosterone therapy. Studies included patients treated with testosterone for symptoms of TD, low serum testosterone levels, and a pathological confirmation of prostate cancer [17]. Their review found that prostate cancer was detected in 7 out of 542 men (1.3%) receiving testosterone therapy and 5 out of 333 men (1.4%) receiving placebo. The authors concluded that there was no evidence that testosterone increased the risk of prostate cancer.

A retrospective study by Ory et al. looked at a cohort of 82 hypogonadal men with prostate cancer treated with testosterone therapy. Median follow-up was 41 months. Prostate-specific antigen increased in patients on active surveillance in this cohort, but no patients were upgraded to a higher Gleason grade on subsequent biopsies [18••].

A retrospective observational case study by Morgentaler et al. examined the experiences of 13 hypogonadal men with biopsy-proven prostate cancer (Gleason grade 6 in 12 men and grade 7 in one man) that were on active surveillance. All men had a follow-up biopsy as part of the protocol. Neither the mean PSA nor prostate volume changes were seen over a 2.5-year median treatment duration. In addition, no prostate cancer progression events were reported [19].

A study from the Netherlands by Debruyne et al. analyzed the Registry of Hypogonadism in Men (RHYME) which is a multinational patient registry of 999 treated and untreated, newly diagnosed hypogonadal men. These patients had baseline and follow-up data that included prostate biopsies, PSA, and testosterone levels. Seventy-five percent (*n* = 750) of men in this study had clinically diagnosed TD and were started on testosterone. A total of 55 biopsies were performed for suspected prostate cancer, and 12 non-cancer-related biopsies were performed. The results of the data showed that the overall proportion of positive biopsies were nearly identical in men on testosterone (37.5%) compared to those not on testosterone (37.0%) for the duration of the study [20••].

In summary, despite the limited data, the available literature concludes that testosterone therapy is safe in men on active surveillance. However, given the lack of prospective, randomized trials, caution should be exercised when initiating therapy.

### Testosterone Therapy in Men After Radical Prostatectomy

Radical prostatectomy (RP) is a common and effective operative approach for the management of prostate cancer. And in the early 2000s, there was mounting clinical evidence that began to fracture the linear mechanistic link between testosterone and prostate cancer. The increasing evidence of the negative health effects of TD and the shift away from testosterone as a prostate cancer inducer have resulted in several studies documenting the role of testosterone therapy in patients who are status post RP.

The first published case series was by Kaufman and Graydon in 2004, who reported on seven men with predominantly low-risk prostate cancer treated with radical prostatectomy with no biochemical recurrences [21]. After surgery, the PSA remained undetectable and continued to be undetectable even after testosterone therapy. The median follow-up time was 2 years. A retrospective study in 2009 by Khera et al. looked at 57 men treated for TD after RP. The entire cohort (predominantly low- and intermediate-risk disease) had negative surgical margins and undetectable serum PSA postoperatively. The cohort was treated with testosterone therapy for an average of 36 months after RP. After a median follow-up time of 1 year, no biochemical recurrences were noted [22].

In 2013, Pastuszak et al. retrospectively reviewed 103 testosterone-deficient men who received testosterone therapy after RP between 2003 and 2011. Also included were 49 eugonadal patients who were also treated with radical prostatectomy but not testosterone as a reference group. The cohort was categorized as being high risk or low risk for postoperative prostate cancer recurrence. Twenty-five percent of the cohort was designated as high risk as defined by a GG > 8 or positive surgical margins or positive lymph nodes. The results showed a statistically significant increase in PSA in the treatment group with a median follow-up of 27.5 months while the no-treatment group showed no detectable increase. However, despite the PSA increase in the treatment group, there were more true prostate cancer recurrences in the control group [23••].

In a 2016 study by Ory et al., a cohort of 82 testosterone-deficient men with prostate cancer, 22 of whom were treated with radical prostatectomy, had their PSA, testosterone, and biochemical recurrence monitored. Of the men treated with RP, none were noted to have a biochemical recurrence [18 ^••^]. The conclusion from these studies seems to show no appreciable increase in prostate cancer recurrence in testosterone-deficient men treated with testosterone after a radical prostatectomy [21, 22, 23••, 24].

### Testosterone Therapy in Men with Prostate Cancer After Radiotherapy

Much like the available published data on testosterone-deficient men with prostate cancer on active surveillance, the data on external beam radiation or brachytherapy for prostate cancer and testosterone therapy is equally sparse. Sarosdy et al. conducted the earliest study in 2007, which was a retrospective review of men with prostate cancer treated using brachytherapy. These patients were subsequently diagnosed with TD and treated with testosterone therapy ranging from 0.5 to 8.5 years (median time 2.0 years). The study reported no biochemical recurrences in this cohort of men with mostly low-risk prostate cancer with a median follow-up time of 5 years [25].

The 2009 study by Morales et al. reported on a series of five men started on testosterone after external beam radiation therapy (EBRT). Patients were on testosterone for an average of 14.5 months with an expected rise in serum testosterone but no evidence of abnormal digital rectal examination; serum PSA remained at <1.1 ng/mL in all patients [26].

The most recent and largest retrospective study to date is by Pastuszak et al. in 2013. This retrospective study looked at 98 men with prostate cancer treated using radiation therapy (either brachytherapy or EBRT) and who were subsequently given testosterone. The median follow-up was 40.8 months and 77% of the cohort had low- or intermediate-risk prostate cancer. There was a clinically insignificant increase in mean PSA with six men meeting criteria for biochemical recurrence during the study period (two of these men requiring initiation of ADT). Two of these patients were treated with brachytherapy for possible biochemical recurrence but may have actually experienced PSA bounce [27••]. The study notes a 6% biochemical recurrence rate, which is lower than previously reported rates status post radiation therapy in the literature. These limited studies on men with prostate cancer status post radiotherapy suggest that testosterone therapy is a safe and effective modality of treatment for TD.

## Conclusion

There has been a critical paradigm in shift in the last 10 years in our understanding of the relationship between androgen levels and prostate cancer. The seminal work by Huggins and Hodges, identifying the effect of androgen blockade on prostate cancer, has forever changed the way we treat prostate cancer. However, misinterpretation of this seminal work has marred the conclusions we have drawn surrounding testosterone therapy in men with prostate cancer.

A large body of evidence in the literature has shown that testosterone deficiency has a significant detrimental effect on health and quality of life that can be mitigated with testosterone therapy. The studies discussed in this review including single- and multi-institution case series, retrospective reviews, and population-based studies have not found a higher than expected risk of prostate cancer progression or recurrence in testosterone-deficient men who received testosterone and were previously treated for prostate cancer (radiotherapy or surgical excision). Table 1 provides a summary of the literature integral to exploring this topic.

**Table 1:** Summary of critical literature for testosterone therapy and prostate cancer

Author	Year	Study design	Patient no.	Treatment type	Results
Calof [15]	2005	Meta-analysis	644	None	Prostate cancer, PSA > 4 ng/mL, and biopsies were higher in the T group than the placebo group.
Sarosdy [24]	2007	Retrospective case study	31	Brachytherapy	No recurrence or progression of prostate cancer (PSA < 1 in all patients)
Shabsigh [16]	2009	Systematic review	2292	Multiple	T therapy did not increase prostate cancer risk or increase Gleason grade in treated vs. untreated men. No consistent effect on PSA
Morgentaler [5]	2011	Retrospective case series	13	Active surveillance	No change in PSA or prostate volume with an increase in mean serum total testosterone. Biopsy in one man and a prostatectomy in another showed no progression or distant disease.
Patuszak [26]	2013	Retrospective case series	13	Radiation therapy	Median f/u of 29.7 months after starting testosterone resulted in large increase in testosterone with no significant increase in PSA. No cancer recurrences in follow-up
Patuszak [22]	2013	Retrospective case series	10	Radical prostatectomy	Median f/u of 27.5 months, significant increases in testosterone and PSA in both high-risk and non-high-risk prostate cancer groups. The reference group had more frequent referrals to radiation oncology or subsequent salvage therapy. There was a significantly increased number of T3b tumors in the reference group vs. testosterone group.
Ory [17]	2015	Retrospective review	82	Active surveillance	PSA increased in patients on active surveillance in this cohort, but no patients were upgraded to a higher Gleason grade on subsequent biopsies.
Millar [27••]	2016	Survey	57	None	The survey showed that 65% of sample of Canadian urologists believe that testosterone therapy is a safe practice. The majority feel that testosterone is safe in post surgical patients; 10–12% fewer are comfortable doing the same in radiated patients. And 20–30% fewer are comfortable with patients on active surveillance.
Debruybe [19]	2017	Retrospective review	999	Multiple	Seventy-five percent of men (750/999) received testosterone therapy. In all, 55 prostate biopsies were performed for prostate cancer. The proportion of prostate biopsies is nearly identical in men on testosterone (37.5%) and those not on testosterone (37.0%).

Despite the data and this paradigm shift, there continues to be significant resistance to the administration of testosterone in men with a history of prostate cancer. A 2016 study by Millar et al. surveyed 56 responding urologists about their practice habits regarding testosterone therapy and prostate cancer. Among those surveyed, 86% actively prescribe testosterone in men with TD, 93% are involved in the treatment of men with prostate cancer, and 95% offer active surveillance. However, only 65% stated they would offer testosterone to men with TD who were on active surveillance for prostate cancer and only 63% believed that testosterone did not increase risk of progression of prostate cancer in these men. Fortunately, 96% believed testosterone was safe for men status post radical prostatectomy and 86% felt it was safe in EBRT and radiation therapy. That said, only 35% of surveyed physicians had ever offered testosterone therapy for men on active surveillance [28••].

With the absence of large randomized placebo-controlled trials, the uncertainty surrounding the safety of testosterone therapy and prostate cancer will remain. However, as Morgentaler has elegantly stated, we have the results of the largest prospective experiment available and that is the natural history of prostate cancer. Prostate cancer is non-existent in men in their 20s when the prostate is bathed in a high testosterone concentration. Rather, the disease becomes more prevalent as men age and testosterone levels decline which more accurately aligns with what we see in the literature.

This review demonstrates that, to date, there is overwhelming evidence that testosterone therapy does not increase prostate cancer risk in the untreated and treated population. This challenges a urological belief that has been cemented in our teaching for over three quarters of a century. We hope that, as we continue to scrutinize the literature in the years to come, we will continue to push forth this paradigm shift.
